# Protection by Ankle Brace for Lower-Extremity Joints in Half-Squat Parachuting Landing With a Backpack

**DOI:** 10.3389/fbioe.2021.790595

**Published:** 2021-12-09

**Authors:** Tianyun Jiang, Shan Tian, Tianhong Chen, Xingyu Fan, Jie Yao, Lizhen Wang

**Affiliations:** ^1^ Key Laboratory of Biomechanics and Mechanobiology (Beihang University), Ministry of Education, Beijing Advanced Innovation Center for Biomedical Engineering, School of Biological Science and Medical Engineering, Beihang University, Beijing, China; ^2^ College of Bioengineering, Chongqing University, Chongqing, China

**Keywords:** ankle brace, lower-extremity joints, backpack, injury prevention, half-squat parachuting landing

## Abstract

Half-squat parachuting landing is a kind of activity with high impact force. Injuries on lower-extremity joints are common in half-squat parachuting landing and would be increased with a backpack. An ankle brace was used to prevent ankle injuries in landing. However, few quantitative studies reported about the protection of an ankle brace for lower-extremity joints in half-squat parachuting landing with a backpack. This study focused on evaluating the protective effects of an ankle brace in half-squat parachuting landing with a backpack. Seven male participants landed from 120 cm with a backpack and an ankle brace. Each participant performed three landing trials on every experimental condition. Kinetics and kinematics of the hip, knee, and ankle were analyzed. It was found that the ankle brace did not significantly affect the ground reaction force with backpack but increased the ground reaction force from 14.7 ± 2.0 bodyweight to 16.2 ± 1.9 bodyweight (*p* = 0.017) without the backpack. The ankle brace significantly (*p* < 0.05) decreased the angular displacement, angular velocity, and angular acceleration of the ankle both without and with the backpack. In conclusion, the ankle brace could restrict ankle motion and significantly increase ground reaction force without the backpack. However, the ankle brace did not significantly influence ground reaction force and still restricted ankle motion with the backpack. Therefore, the ankle brace was more effective in half-squat parachuting landing with the backpack than no-backpack landing.

## Introduction

Parachuting landing, one kind of landing with high impact force, was common in military and civil activities. Injuries, such as ankle fracture, ankle sprains, and hip contusions, occurred on lower-extremity joints (i.e., hip, knee, and ankle) due to the high impact force on the lower extremity (Ekeland, 1997; Knapik et al., 2011; Zakowski et al., 2019). Half-squat parachuting landing is one kind of parachuting landing in China, in which the left and right knees and ankles hug each other and the feet are in parallel with the ground (Niu et al., 2010). Injuries on the lower-extremity joints were commonly seen in the half-squat parachuting landing (Li et al., 2013). This study would focus on decreasing injuries of the lower-extremity joints in the half-squat parachuting landing.

An ankle brace was developed to decrease injuries in parachuting landing by restricting the excessive motion of the ankle (Knapik et al., 2008; Knapik and Steelman, 2016; Tamura et al., 2017). It was reported that inversion ankle sprains were decreased by the ankle brace from 0.379% to 0.055% (Schmidt et al., 2005). Kinetic and kinematic parameters of the lower-extremity joints, including ground reaction force, joint moment, joint energy absorption, angular displacement, angular velocity, and angular acceleration, were used as indicators in injury evaluation of the lower-extremity joints and the protective effects of ankle brace (Gardner et al., 2012; Tamura et al., 2016; Wu et al., 2018; Sato et al., 2019).

The ankle brace, on one hand, could restrict ankle motion, decrease the angular displacement and the angular velocity of the ankle, and increase stabilization of the ankle in the half-squat parachuting landing (Niu et al., 2011; Wu et al., 2018). However, on the other hand, the ankle brace could also increase the ground reaction force in the half-squat parachuting landing (Niu et al., 2011), which was the negative effect of the ankle brace protection. In the drop landing, the joint moment of the ankle was not significantly influenced with the ankle brace (Zhang et al., 2012; Maeda et al., 2019). The joint energy absorption of the ankle was decreased by the ankle brace (Gardner et al., 2012). The angular displacement of the ankle was also decreased by the ankle brace (Cordova et al., 2010; Zhang et al., 2012; Kuni et al., 2016; Mason-Mackay et al., 2016). However, in the half-squat parachuting landing, the effects of an ankle brace on the joint moment, joint energy absorption, and angular acceleration of the ankle were not clear.

In our previous study, the multi-joint protection for the hip, knee, and ankle in the half-squat parachuting landing was found to be provided by a knee brace, which was another type of protective device (Jiang et al., 2020). For motion of the knee and hip in the drop landing, it was reported that the angular displacement of the knee and the hip was not significantly influenced by the ankle brace in the previous study (Cordova et al., 2010; Agres et al., 2019; Maeda et al., 2019). It was meaningful for protecting the lower-extremity joints if an ankle brace could provide the multi-joint protection for the hip, knee, and ankle. However, in the half-squat parachuting landing, there were few studies on whether an ankle brace could provide the multi-joint protection for the hip, knee, and ankle. This study would analyze kinetics and kinematics of the hip, knee, and ankle in the half-squat parachuting landing to evaluate the protective effects of an ankle brace.

A backpack, containing the necessities for military missions and living, was carried in parachuting landing, which would increase injuries by approximate 160% (Knapik and Steelman, 2016). Ankle sprain and ankle fracture were increased by 71% and 179% by the backpack, respectively (Knapik et al., 2008). In the half-squat parachuting landing with a backpack, there were a few studies about injury evaluation. In the drop landing with a 15 kg backpack, the peak vertical ground reaction force was significantly increased by 0.27 bodyweight (BW) (Sell et al., 2010). It was suggested that a backpack could increase the impact force and injuries of the lower-extremity joints. However, there were few studies about the protective effects of an ankle brace on the hip, knee, and ankle in half-squat parachuting landing with a backpack.

The purpose of this study was to evaluate the protective effects of an ankle brace on lower-extremity joints in half-squat parachuting landing with a backpack by analyzing kinetics and kinematics of the hip, knee, and ankle. Participants would be recruited for the experiment. The hypothesis was that the ankle brace could protect the lower-extremity joints in the half-squat parachuting landing with the backpack.

## Materials and Methods

### Participants

Seven participants (male; 22.0 ± 3.0 years old; 176.2 ± 4.2 cm height; 67.6 ± 6.2 kg weight; no injury history since 1 year ago) were recruited. The studies involving human participants were reviewed and approved by the Science and Ethics Committee of School of Biological Science and Medical Engineering in Beihang University, China (no. BM201900121). The participants provided their written informed consent to participate in this study.

### Equipment

**FIGURE 1:**
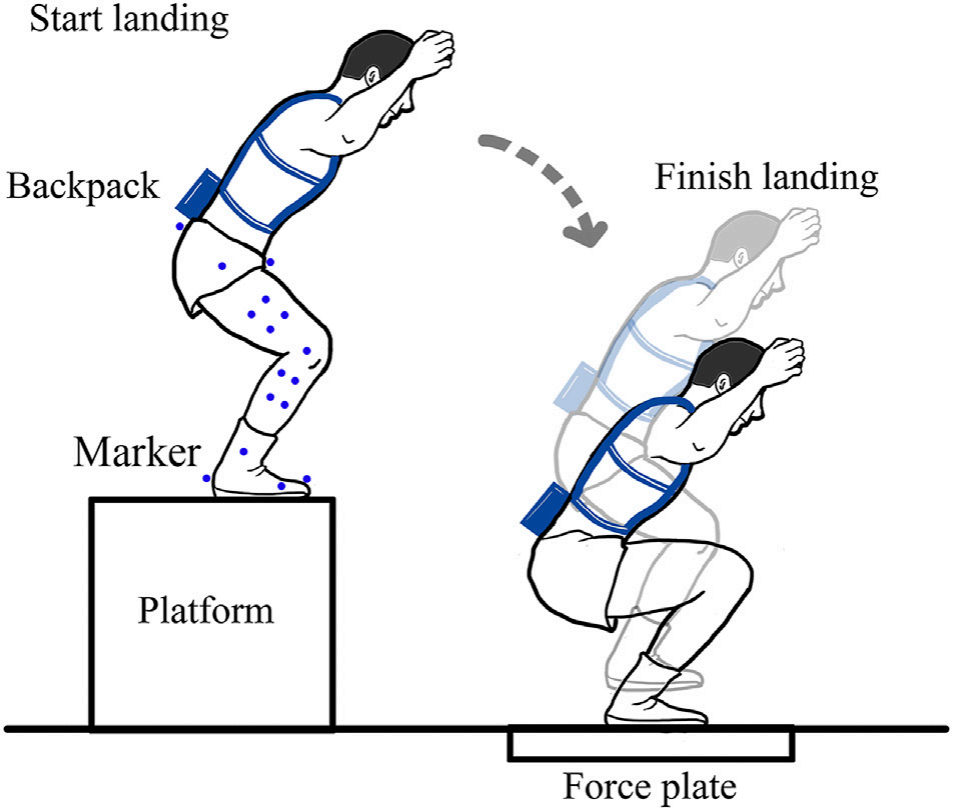
The schedule of the half-squat parachuting landing experiment.

Thirteen passive infrared reflex markers were attached to the bony landmarks of the participant: the left anterior superior iliac spines, the left posterior superior iliac spines, the right anterior superior iliac spines, the right posterior superior iliac spines, the greater trochanter, the lateral knee, the medial knee, the lateral ankle, the medial ankle, the heel, the fifth metatarsal, the second toe, and the first toe, as shown in Figure 1. Eight markers were attached to the surface of the thigh and the shank, respectively.

**FIGURE 2:**
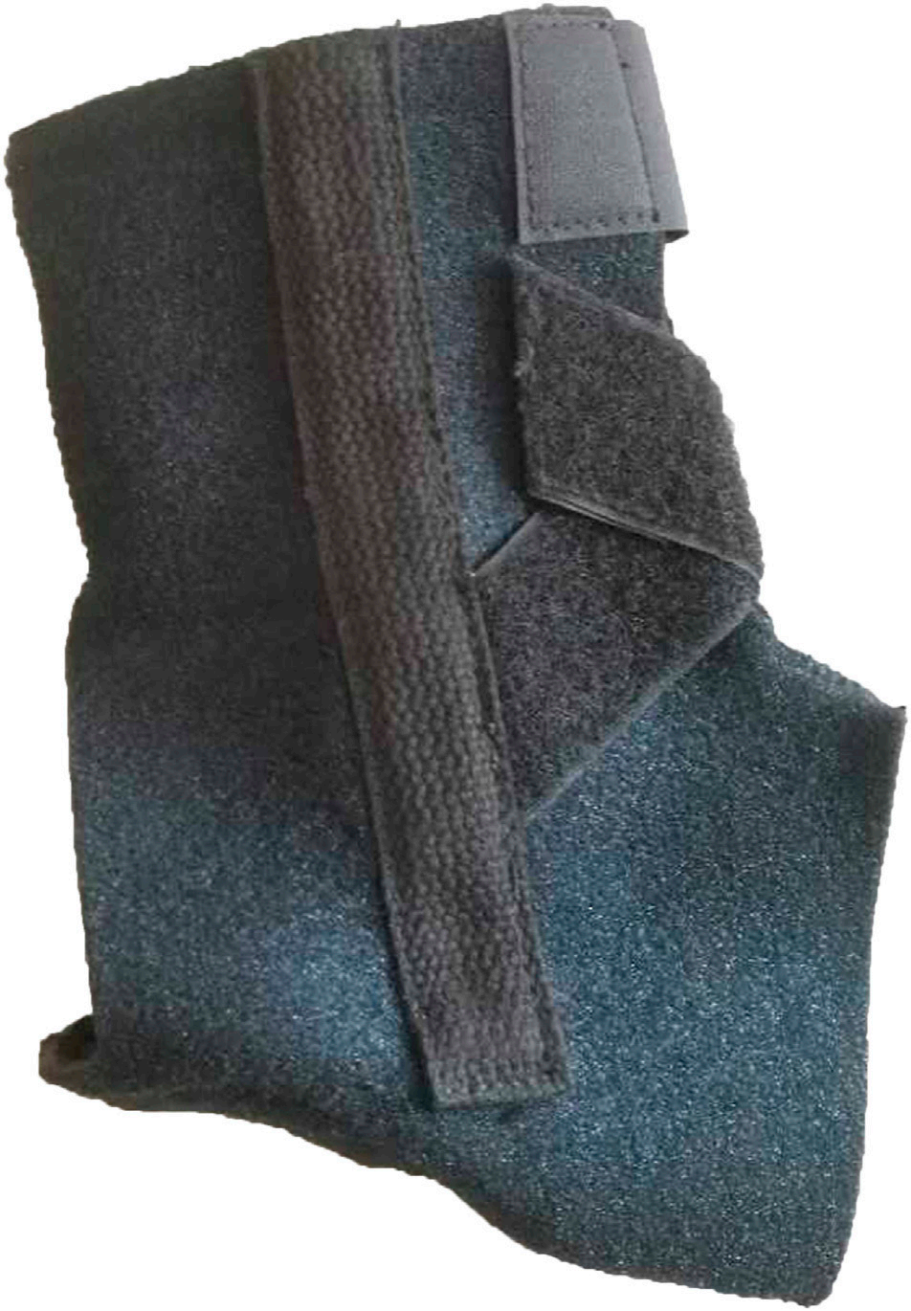
The ankle brace in this study.

A 5 kg sports vest was regarded as the backpack because the markers on the pelvis could be conveniently attached and traded. The ankle brace in this study was made of terylene, spandex, and elastic fiber, as shown in Figure 2. A bar made of aluminum alloy and a spring bar made of no. 72A spring steel were put into two sides of the ankle brace near the lateral ankle and the medial ankle, respectively. The two bars were shaped to be suitable for the anatomic structure of the lateral ankle and the medial ankle, respectively. A soft pad was placed on the bottom of the ankle brace under the heel to improve comfort.

### Procedure

The experimental conditions were group 1: no backpack + no brace (without the backpack and without the ankle brace), group 2: backpack + no brace (with the backpack and without the ankle brace), group 3: no backpack+ ankle brace (without the backpack and with the ankle brace), and group 4: backpack+ ankle brace (with the backpack and with the ankle brace). The three-dimensional displacements of the markers were measured by using a three-dimensional motion capture system (VICON, Oxford Metrics, UK) at the sample frequency of 200 Hz. The ground reaction force was measured by using the force plate (900 mm × 600 mm× 100 mm, AMTI, United States) at the sample frequency of 1,600 Hz. All participants had already trained the half-squat parachuting landing technique before trials. Before landing, the participant stood straightly and unbent two arms with the palms toward the body to get the static calibration of the markers. Then, the participant dropped from a 120 cm high platform and landed on the force plate, as shown in Figure 1. Each participant performed three trials for each experimental condition.

### Data Analysis

A multi-rigid-body model including the pelvis and right lower-extremity was developed based on the static calibration of markers by using the Visual3D software (C-Motion Inc., United States). The center of the hip was estimated based on the markers on the left anterior superior iliac spines, the left posterior superior iliac spines, the right anterior superior iliac spines, the right posterior superior iliac spines, and the greater trochanter. The center of the knee was defined as the middle point between the markers on the lateral and medial knee. The center of the ankle was defined as the middle point between the markers on the lateral and medial ankle.

The angular displacement and the joint moment of the hip, knee, and ankle on sagittal plane and frontal plane were computed by using the Visual3D software. The angular velocity and angular acceleration of the hip, knee, and ankle on sagittal plane and frontal plane were computed based on the angular displacement and the numerical differential formula. The joint energy absorption was computed by integrating the joint moment over the angular displacement. The ground reaction force, the joint moment, and the joint energy absorption were normalized with BW.

The peak vertical ground reaction force and the joint moment, the joint energy absorption, the angular displacement, the angular velocity, and the angular acceleration of the hip, knee, and ankle on sagittal plane and frontal plane were analyzed by using ANOVA by SPSS v19.0 software. The level of significant difference was *p* < 0.05. The study power was analyzed using G*Power based on the data of angular displacement of the knee, assuming an alpha of 0.05. The statistical power (1-beta) was equal to 1.0, larger than 0.8. The Kolmogorov–Smirnov method was used to test that all outcomes conformed to normal distribution. The one-way repeated measures ANOVA was used to test the repeatability of the three trials based on the angular displacement of the hip, knee, and ankle on sagittal plane and the peak ground reaction force in the four experimental groups, and there was a good repeatability of the three trials (*p* > 0.05).

## Results

### Kinetics

The peak vertical ground reaction force was increased by the backpack from 14.7 ± 2.0 to 16.0 ± 1.6BW (*p* = 0.012), as shown in Table 1. The peak vertical ground reaction force was significantly increased from 14.7 ± 2.0 to 16.2 ± 1.9 BW (*p* = 0.017) by the ankle brace in the landing without the backpack. There was a significant difference in the interaction (*p* = 0.009) between the backpack and the ankle brace in the peak vertical ground reaction force.

**TABLE 1:** The peak vertical ground reaction force, the joint moment, and the joint energy absorption.

		Group 1: no backpack + no brace	Group 2: backpack + no brace	Group 3: no backpack + ankle brace	Group 4: backpack + ankle brace
Peak vertical ground reaction force (BW)^a^ ^,^ ^b^		14.7 ± 2.0	16.0 ± 1.6	16.2 ± 1.9	15.3 ± 2.0
Joint moment on sagittal plane (BW)	Hip	1.77 ± 0.51	1.67 ± 0.50	1.97 ± 0.42	1.58 ± 0.36
	Knee	0.86 ± 0.27	0.84 ± 0.22	0.89 ± 0.54	0.73 ± 0.18
	Ankle	0.36 ± 0.11	0.31 ± 0.08	0.37 ± 0.11	0.29 ± 0.07
Joint moment on frontal plane (BW)	Hip	0.62 ± 0.18	0.59 ± 0.27	0.69 ± 0.17	0.56 ± 0.18
	Knee	0.31 ± 0.08	0.28 ± 0.13	0.35 ± 0.18	0.24 ± 0.08
	Ankle	0.11 ± 0.08	0.09 ± 0.03	0.11 ± 0.03	0.08 ± 0.03
Joint energy absorption (BW)	Hip^a^	0.11 ± 0.04	0.14 ± 0.05	0.12 ± 0.02	0.13 ± 0.04
	Knee^a^	0.41 ± 0.07	0.49 ± 0.07	0.45 ± 0.19	0.47 ± 0.06
	Ankle^a^ ^,^ ^b^ ^,^ ^c^	0.10 ± 0.02	0.11 ± 0.02	0.08 ± 0.03	0.09 ± 0.02

a Significant difference (*p* < 0.05) between group 1 and group 2.

b Significant difference (*p* < 0.05) between group 1 and group 3.

c Significant difference (*p* < 0.05) between group 2 and group 4.

The joint moment of the hip, knee, and ankle on sagittal plane and frontal plane was not significantly affected (*p* > 0.05) with the backpack as well as the ankle brace, as shown in Table 1. There were no significant differences in the interaction (*p* > 0.05) between the backpack and the ankle brace in the joint moment of the hip, knee, and ankle on sagittal plane and frontal plane.

As shown in Table 1, the joint energy absorption of the hip, knee, and ankle was increased by the backpack from 0.11 ± 0.04 BW, 0.41 ± 0.07 BW and 0.10 ± 0.02 BW to 0.14 ± 0.05 BW (*p* = 0.037), 0.49 ± 0.07 BW (*p* = 0.001) and 0.11 ± 0.02 BW (*p* = 0.045) respectively. The joint energy absorption of ankle was decreased by the ankle brace from 0.10 ± 0.02 BW to 0.08 ± 0.03 BW (*p* = 0.037) without the backpack, and was decreased from 0.11 ± 0.02 BW to 0.09 ± 0.02 BW (*p* < 0.001) with the backpack. There was no significant difference in the interaction (*p* > 0.05) between the backpack and the ankle brace in the joint energy absorption of the hip, knee, and ankle.

### Kinematics

As shown in Table 2, the angular displacement of the hip, knee, and ankle on sagittal plane was increased from 52.6 ± 7.2°, 102.1 ± 9.3° and 45.2 ± 7.8° to 60.1 ± 8.2° (*p* = 0.006), 110.1 ± 9.3° (*p* = 0.009) and 53.8 ± 5.1° (*p* < 0.001) respectively by the backpack. The angular displacement of ankle on sagittal plane was decreased from 45.2 ± 7.8° to 36.0 ± 10.9° (*p* = 0.005) by the ankle brace without the backpack, and was decreased from 53.8 ± 5.1° to 48.0 ± 5.5° (*p* = 0.001) by the ankle brace with the backpack. There was no significant difference in the interaction (*p* > 0.05) between the backpack and the ankle brace in the angular displacement of the hip, knee, and ankle on sagittal plane.

**TABLE 2:** The angular displacement, the angular velocity, and the angular acceleration.

		Group 1: no backpack ± no brace	Group 2: backpack + no brace	Group 3: no backpack + ankle brace	Group 4: backpack + ankle brace
Angular displacement on sagittal plane (°)	Hip^a^	52.6 ± 7.2	60.1 ± 8.2	53.6 ± 9.6	65.1 ± 9.1
	Knee^a^	102.1 ± 9.3	110.1 ± 9.3	104.2 ± 17.6	105.3 ± 16.0
	Ankle^a^ ^,^ ^b^	45.2 ± 7.8	53.8 ± 5.1	36.0 ± 10.9	48.0 ± 5.5
Angular velocity on sagittal plane (°/s)	Hip^a^	438.9 ± 71.4	482.2 ± 54.1	410.0 ± 90.0	483.4 ± 101.3
	Knee^a^	808.9 ± 54.4	855.9 ± 65.3	795.1 ± 78.0	865.0 ± 79.4
	Ankle^a^ ^,^ ^b^	508.7 ± 105.9	594.0 ± 99.5	332.0 ± 104.2	471.3 ± 110.4
Angular acceleration on sagittal plane (°/s^2^)	Hip^a^	8,165.9 ± 1,920.6	9,810.8 ± 1,478.1	7,605.9 ± 2,029.0	10,506.6 ± 1,920.6
	Knee^a^	12,791.7 ± 1,266.5	14,025.1 ± 1,979.6	13,151.5 ± 2,637.2	13,327.7 ± 1,407.1
	Ankle^a^ ^,^ ^b^	10,813.6 ± 2,179.3	13,099.7 ± 2,280.0	7,764.4 ± 2,542.5	11,077.1 ± 1,703.4
Angular displacement on frontal plane (°)	Hip	6.9 ± 3.3	8.2 ± 3.7	7.6 ± 2.5	8.2 ± 3.8
	Knee	12.5 ± 2.4	14.5 ± 2.3	9.9 ± 2.3	13.0 ± 3.8
	Ankle^b^	13.1 ± 3.6	14.5 ± 4.7	9.4 ± 4.8	11.5 ± 3.5
Angular velocity on frontal plane (°/s)	Hip	67.6 ± 15.8	69.6 ± 17.0	59.8 ± 15.1	72.7 ± 13.9
	Knee	165.5 ± 36.1	181.3 ± 42.4	131.1 ± 40.2	167.3 ± 34.4
	Ankle^b^	159.4 ± 37.5	180.5 ± 56.1	115.9 ± 48.6	151.7 ± 30.1
Angular acceleration on frontal plane (°/s^2^)	Hip	2,061.5 ± 636.0	2,182.7 ± 558.1	2,068.1 ± 574.4	2,186.7 ± 511.5
	Knee	5,271.0 ± 1,424.5	5,195.0 ± 1,042.7	5,985.3 ± 1,228.7	5,534.2 ± 1,468.0
	Ankle^b^	3,895.2 ± 792.1	3,997.8 ± 670.6	3,075.4 ± 972.4	3,515.3 ± 738.8

a Significant difference (*p* < 0.05) between group 1 and group 2.

b Significant difference (*p* < 0.05) between group 1 and group 3 and between group 2 and group 4.

As shown in Table 2, the angular velocity of the hip, knee, and ankle on sagittal plane was increased from 438.9 ± 71.4°/s, 808.9 ± 54.4°/s, and 508.7 ± 105.9°/s to 482.2 ± 54.1°/s (*p* = 0.037), 855.9 ± 65.3°/s (*p* = 0.022), and 594.0 ± 99.5°/s (*p* = 0.014), respectively, by the backpack. The angular velocity of the ankle on sagittal plane was decreased from 508.7 ± 105.9°/s to 332.0 ± 104.2°/s (*p* < 0.001) by the ankle brace without the backpack and was decreased from 594.0 ± 99.5°/s to 471.3 ± 110.4°/s (*p* = 0.001) by the ankle brace with the backpack. There were no significant differences in the interaction (*p* > 0.05) between the backpack and the ankle brace in the angular velocity of the hip, knee, and ankle on sagittal plane.

As shown in Table 2, the angular acceleration of the hip, knee, and ankle on sagittal plane was increased from 8,165.9 ± 1,920.6°/s^2^, 12,791.7 ± 1,266.5°/s^2^, and 10,813.6 ± 2,179.3°/s^2^ to 9,810.8 ± 1,478.1°/s^2^ (*p* = 0.003), 14,025.1 ± 1,979.6°/s^2^ (*p* = 0.021), and 13,099.7 ± 2,280.0°/s^2^ (*p* = 0.002), respectively, by the backpack. The angular acceleration of the ankle on sagittal plane was decreased from 10,813.6 ± 2,179.3°/s^2^ to 7,764.4 ± 2,542.5°/s^2^ (*p* < 0.001) by the ankle brace without the backpack and was decreased from 13,099.7 ± 2,280.0°/s^2^ to 11,077.1 ± 1,703.4°/s^2^ (*p* = 0.002) by the ankle brace with the backpack. There were no significant differences in the interaction (*p* > 0.05) between the backpack and the ankle brace in the angular acceleration of the hip, knee, and ankle on sagittal plane.

As shown in Table 2, the angular displacement of the ankle on frontal plane was decreased from 13.1 ± 3.6° to 9.4 ± 4.8° (*p* = 0.017) by the ankle brace without the backpack and was decreased from 14.5 ± 4.7° to 11.5 ± 3.5° (*p* = 0.026) by the ankle brace with the backpack. There were no significant differences in the interaction (*p* > 0.05) between the backpack and the ankle brace in the angular displacement of the hip, knee, and ankle on frontal plane.

As shown in Table 2, the angular velocity of the ankle on frontal plane was decreased from 159.4 ± 37.5°/s to 115.9 ± 48.6°/s (*p* = 0.005) by the ankle brace without the backpack and was decreased from 180.5 ± 56.1°/s to 151.7 ± 30.1°/s (*p* = 0.043) by the ankle brace with the backpack. There were no significant differences in the interaction (*p* > 0.05) between the backpack and the ankle brace in the angular velocity of the hip, knee, and ankle on frontal plane.

As shown in Table 2, the angular acceleration of the ankle on frontal plane was decreased from 3,895.2 ± 792.1°/s^2^ to 3,075.4 ± 972.4°/s^2^ (*p* = 0.009) by the ankle brace without the backpack and was decreased from 3,997.8 ± 670.6°/s^2^ to 3,515.3 ± 738.8°/s^2^ (*p* = 0.032) by the ankle brace with the backpack. There were no significant differences in the interaction (*p* > 0.05) between the backpack and the ankle brace in the angular acceleration of the hip, knee, and ankle on frontal plane.

## Discussion

In this study, kinetic and kinematic parameters of the hip, knee, and ankle were analyzed to evaluate the protective effects of an ankle brace for the lower-extremity joints in the half-squat parachuting landing with a backpack. It was found that the ankle brace could provide more effective protection in the half-squat parachuting landing with the backpack than that without the backpack.

### The Effects of the Backpack

The peak vertical ground reaction force was increased by the backpack, as shown in Table 1. The joint energy absorption of the hip, knee, and ankle was significantly increased with the backpack, as shown in Table 1. These were because the additional weight and the gravity potential energy of the participant were increased by the backpack. Similar results in the drop landing with the backpack were shown in a previous study, in which the peak vertical ground reaction force was increased, and the increase of the ground reaction force indicated more injuries of the lower extremity (Sell et al., 2010). The higher ground reaction force could induce higher angular acceleration of the hip, knee, and ankle (Table 2). Therefore, one purpose of designing an ankle brace was to decrease the ground reaction force. With the backpack, the lower-extremity joints absorbed more impact energy to prevent injuries, which was the instinct of body. The knee absorbed more impact energy than the hip and ankle, which was the same as the results in a previous study (Lee et al., 2018).

**FIGURE 3:**
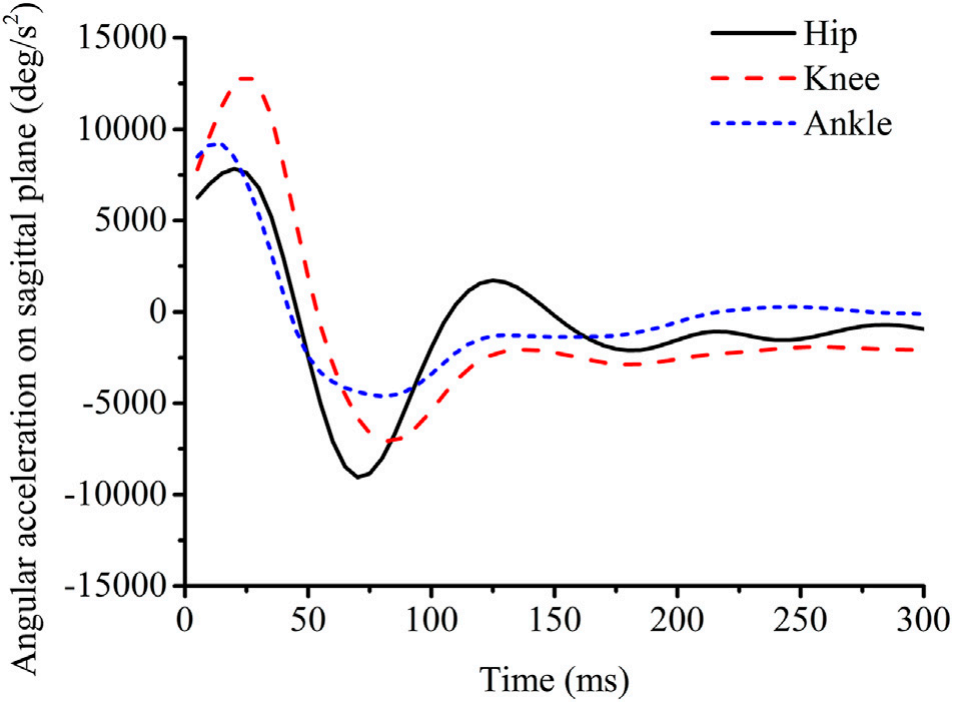
Angular acceleration of the hip, knee, and ankle on sagittal plane.

The joint moment of the hip, knee, and ankle was not significantly affected by the backpack, as shown in Table 1. The angular displacement and the angular velocity of the hip, knee, and ankle on sagittal plane were significantly increased with the backpack, as shown in Table 2. A similar outcome was reported that the angular displacement of the knee was increased with the backpack in the drop landing (Sell et al., 2010). The increase of the angular displacement of lower-extremity joints on sagittal plane would decrease the landing stabilization and increase injuries (Wu et al., 2017). With the higher angular velocity, stress on soft tissues of the joints might be increased due to the viscoelasticity of the soft tissues (Jiang et al., 2020). The increase of the angular velocity could increase injuries of the joints. The higher angular acceleration of the knee was also an indicator of the non-contact injury of the anterior cruciate ligament in single-leg drop landing (Tamura et al., 2016). Therefore, designing an ankle brace should decrease angular displacement, angular velocity, and angular acceleration. It could be found that there were two directions of angular acceleration on sagittal plane: positive direction, which is the same to the direction of angular displacement and angular velocity, and negative direction (Figure 3). The positive direction represented that the impact force flexed the joints, while the negative direction represented that lower-extremity joints were counterbalanced by the muscle activation of the lower extremity to keep stability and absorb the impact energy. No significances in the joint moment, angular displacement, angular velocity, and angular acceleration of the hip, knee, and ankle on frontal plane were found with the backpack, as shown in Tables 1 and 2. The half-squat parachuting landing posture required that the left and right knees and ankles hugged each other, and the joints’ motion on the coronal plane might be restricted.

### The Effects of the Ankle Brace

The peak vertical ground reaction force was increased with the ankle brace in the landing without the backpack (Table 1), possibly because the joint energy absorption of the lower-extremity joints was significantly decreased with the ankle brace (Table 1). Similar results were reported in a previous study that the peak vertical ground reaction force was increased by the ankle brace in the half-squat parachuting landing (Niu et al., 2011). The increase in the ground reaction force could increase injuries, which was the negative effect of the ankle brace because it increased the ground reaction force. The protective effect of the ankle brace could be decreased. However, the negative effect of the ankle brace was lost in the half-squat parachuting landing with the backpack. With the backpack, the ground reaction force was not significantly influenced by the ankle brace (Table 1). The reason might be that the impact energy was absorbed by other joint motions of the body such as trunk flexion, except for the lower-extremity joint motion in the landing with the backpack. It could be suggested that the ankle brace could provide better protection in the half-squat parachuting landing with the backpack than without the backpack. For the half-squat parachuting landing in military area, a backpack was commonly carried. It was meaningful to use an ankle brace in the half-squat parachuting landing especially with a backpack.

The joint moments of the hip, knee, and ankle were not significantly influenced by the ankle brace in the landing both without and with the backpack. This outcome was similar to that in the drop landing (Zhang et al., 2012). The angular displacement, the angular velocity, and the angular acceleration of the ankle on sagittal plane were decreased with the ankle brace in the landing both without and with the backpack, as shown in Table 2. The outcomes in this study were satisfactory for protection. The ankle brace could still restrict ankle motion and provide protective effects for the ankle without the backpack, even though there was as negative effect that the ankle brace increased the ground reaction force without the backpack as shown in Table 1. The angular displacements and the angular velocities of the hip and knee on sagittal plane were not significantly influenced with the ankle brace, as shown in Table 2. A similar outcome was reported that the ankle brace did not significantly influence the angular displacement of the hip on sagittal plane in the drop landing (Cordova et al., 2010). One plausible reason was that the ankle brace did not restrict the angular displacement and the angular velocity of the ankle sufficiently enough to significantly influence the angular displacement and the angular velocity of the hip and knee (Simpson et al., 2013).

The motion of the ankle on frontal plane was another factor of ankle sprain injury (Niu et al., 2011). Injuries of the ankle could be induced by greater angular displacement and angular velocity of the ankle on frontal plane (Bhaskaran et al., 2015; Sinsurin et al., 2017; Hanzlikova et al., 2019). Ankle sprain would be prevented by the decrease in the angular displacement of the ankle on frontal plane (Klem et al., 2017). The angular displacement, the angular velocity, and the angular acceleration of the ankle on frontal plane were decreased by the ankle brace, as shown in Table 2. The ankle motion on frontal plane was restricted by the ankle brace, which would even benefit users whose ligaments on the ankle have been injured before (Choisne et al., 2019).

### Multi-Joint Protection

In this study, the ankle brace did not significantly influence the ground reaction force with the backpack but increased the ground reaction force without the backpack. In our previous study, the multi-joint protection of a knee brace for the hip, knee, and ankle was provided in the half-squat parachuting landing without the backpack, but the knee brace could only protect the knee in the half-squat parachuting landing with the backpack (Jiang et al., 2020). Different from the knee brace, the ankle brace could not provide multi-joint protection because the ankle brace could only protect the ankle in the half-squat parachuting landing both without and with the backpack. However, it was not suggested that the ankle brace was useless. In the real-life half-squat parachuting landing, paratroopers commonly carried heavy backpacks. The injury risk of the ankle was 36%, higher than that of the knee (18%) (Ekeland, 1997). The knee brace could not protect the ankle in the half-squat parachuting landing with the backpack. Therefore, the ankle brace was necessary for ankle protection.

### Limitations

One limitation of this study was that the sample size of the participants was limited due to the cost. Each participant performed three trials to enlarge the sample size. The G*Power software was used to analyze the study power of the sample size. Only male participants were recruited in this experiment because the injury risk of male paratroopers was higher than that of females (Fer et al., 2021). Another limitation was that the experimental design could not fully simulate the real-life half-squat parachuting landing. The backpack load was lighter than the real weight due to safety. Besides, it was hard to use the real backpack, which would shade markers on the pelvis, and there were few experimental studies on the real backpack. Even with these limitations, we still found a trend that the backpack affected the kinematic and kinetic parameters of the hip, knee, and ankle.

## Conclusion

The ankle brace could restrict ankle motion and protect the ankle in the half-squat parachuting landing both without and with the backpack. The ankle brace significantly increased the ground reaction force, which thus decreased the protective effects of the ankle brace. The ankle brace did not significantly increase the ground reaction force but could still maintain the protective effects with a backpack. The ankle brace could provide more effective protection for the lower-extremity joints in the half-squat parachuting landing with a backpack than no-backpack landing.

## Data Availability

The original contributions presented in the study are included in the article/Supplementary Material; further inquiries can be directed to the corresponding author.
